# Increased Cancer Risk in Younger Patients with Thyroid Nodules Diagnosed as Atypia of Undetermined Significance

**DOI:** 10.7759/cureus.2348

**Published:** 2018-03-19

**Authors:** Emilija Todorovic, Brandon S Sheffield, Steve Kalloger, Blair Walker, Sam M Wiseman

**Affiliations:** 1 Pathology and Laboratory Medicine, St. Paul’s Hospital & University of British Columbia; 2 Pathology and Laboratory Medicine, Vancouver General Hospital & University of British Columbia; 3 Surgery, St. Paul’s Hospital & University of British Columbia

**Keywords:** thyroid nodule, bethesda system, atypia of undetermined significance, follicular lesion of undetermined significance, cytology, fine needle aspiration biopsy

## Abstract

Background: The objective of this study was to determine if patient age and/or gender significantly alter the risk of thyroid malignancy in the Bethesda System for Reporting Thyroid Cytopathology (BSRTC) diagnostic categories.

Methods: A retrospective review of 291 sequential patients that underwent thyroid nodule fine needle aspiration biopsy (FNAB) and subsequent surgery at a single center was carried out. Cases were grouped according to age (55 years and older versus younger than 55 years) and gender. The cancer risk was calculated for each BSRTC diagnostic group. A p-value <0.05 was not considered statistically significant.

Results: The study population was composed of 291 patients (227 females and 64 males). Histopathology diagnosed cancer in 113 cases (39%). The cancer risk was significantly increased in cases with a BSRTC diagnosis of atypia of undetermined significance/follicular lesion of undetermined significance (AUS/FLUS) in patients younger than 55 years of age (36.8% vs 7.4%, p=0.0082).

Conclusions: Though thyroid cancer was significantly more common in males (p=0.021), gender did not significantly influence specific BRSTC diagnostic category cancer risk estimation. A BSRTC AUS/FLUS diagnosis is associated with an increased cancer risk in younger patients.

## Introduction

For more than 20 years, the incidence of thyroid cancer has steadily increased globally [1], exhibiting the most rapid rise of all major human cancer types. There is a strong suggestion that it is the increased detection of papillary carcinomas (PTCs), especially small PTCs, that is primarily responsible for this rising incidence [2]. Cancer of the thyroid gland usually presents as a nodule, and thyroid nodules may either represent the presenting complaint or be an incidental finding. Fortunately, the majority (95%) of all thyroid nodules are benign. Nonetheless, thyroid nodule detection raises concerns regarding the possibility of an underlying malignancy and usually requires further investigation. The gold standard for thyroid nodule investigation is fine needle aspiration biopsy (FNAB). It is a critically important diagnostic method for distinguishing benign from malignant nodules and guiding their further treatment [3]. In 2007, in order to reduce variation between pathologists in the reporting of cytopathological findings, and also to standardize diagnostic terminology, the National Cancer Institute held a consensus conference and developed recommendations for standardized reporting of thyroid cytopathological diagnoses. Having been named after the location of this conference, these thyroid cytopathology reporting guidelines have become known as the Bethesda System for Reporting Thyroid Cytopathology (BSRTC) [4]. As stated in the 2015 American Thyroid Association (ATA) Management Guidelines for Adult Patients with Thyroid Nodules and Differentiated Thyroid Cancer, it is now a strong recommendation that all centers utilize the BSRTC for the reporting of thyroid nodule cytopathology [5]. The BSRTC is a six-tiered classification system, with each diagnostic category having a specific associated risk of malignancy (ROM), as well as recommended management. These six BSRTC diagnostic categories are: unsatisfactory, benign, atypia of undetermined significance/follicular lesion of undetermined significance (AUS/FLUS), suspicious for follicular neoplasm/follicular neoplasm (sFN), suspicious for malignancy (sM), and malignant. The quoted ROM for each of these six BSRTC diagnostic categories are: 1%–4%, 0%–3%, 5%–15%, 15%–30%, 60%–75% and 97%–99%, respectively [4].

The most challenging BSRTC diagnostic categories for the clinician to manage are the three indeterminate categories (AUS/FLUS, sFN and sM). Due to an associated low cancer risk, it is the AUS/FLUS diagnostic group that is especially difficult to manage. Despite the recommendations that AUS/FLUS be used as a ‘last resort’ diagnostic category, and that it should represent no greater than 7% of all BSRTC diagnoses at a particular institution [4], there is considerable variability in its utilization at different centers, with reporting rates that range from 0.8% to 27.12% [6]. Several studies have also reported a higher ROM for the AUS/FLUS diagnostic category than has been previously suggested [3, 7]. In the current ATA guidelines, for thyroid nodules diagnosed as AUS/FLUS, in addition to a repeat FNAB, a multidisciplinary approach that combines clinical, sonographic and molecular characteristics, is now recommended [5]. While many of the variables that are taken into account to assist with making clinical decisions are subjected to confounding influences, patient variables such as age and gender remain consistent.

Thyroid cancer is more commonly diagnosed in younger people and in women [4]. Unlike most other human cancer types, age represents an important disease prognosticator for individuals diagnosed with differentiated thyroid cancer (DTC). In the latest American Joint Commission on Cancer (AJCC) thyroid cancer staging system [8], cancer in patients 55 years and older is generally associated with a more aggressive clinical course and worse prognosis. Very recently, following large-scale studies, the 45-year age cut-off for cancer staging, and thus disease prognostication for differentiated thyroid cancers has been raised to 55 years [8-9]. Studies have shown that this older age cut-off improves the accuracy of the prognostication system, and therefore should help prevent overtreatment of patients. The objective of this study was to review the distribution of the BSRTC diagnostic categories at our institution and to determine if patient age or gender significantly modifies the risk of malignancy in the indeterminate (AUS/FLUS, sFN, and sM) BSRTC diagnostic groups.

## Materials and methods

A retrospective chart and pathology review of consecutive patients at our center that underwent thyroid nodule FNAB and a subsequent thyroid operation was carried out. The final histopathological diagnoses of the thyroidectomy specimens were considered to be the gold standard for nodule diagnosis. To ensure consistency within the study with respect to cytopathological and histopathological evaluations, all cases that underwent thyroid FNAB and/or operation at other institutions were excluded from the study population. All cases of papillary thyroid microcarcinoma (defined as papillary carcinoma smaller than 1 cm in maximum diameter) that were not an index lesion of interest, and thus were incidentally diagnosed by histopathology, were grouped with the benign cases for the analysis. The study population was then grouped according to age (younger than 55 years or 55 years and older) and gender. The cytology slides were evaluated by one or more board-certified pathologists at our center. The FNABs were classified based upon their BSRTC diagnoses, and they were reported by the pathologists as being either unsatisfactory, benign, AUS/FLUS, sFN, sM, or malignant. For cases that underwent multiple FNABs in the current study, the diagnosis of AUS/FLUS was based upon the FNAB that most immediately preceded the thyroid operation. Though influenced by clinical factors, it is common practice at our institution to repeat the FNAB if the initial diagnosis is either unsatisfactory or AUS/FLUS. All cases not reported according to the BSRTC were excluded from the study population. The characteristics of the study population are summarized in Table 1. A ROM for each BSRTC diagnostic category was calculated after grouping cases by either their age or gender. Statistical analyses that compared the ROM between categories were calculated using the Fisher exact test. A p-value less than 0.05 was considered to be statistically significant.

**Table 1:** Distribution of fine needle aspiration biopsies (FNABs) according to the Bethesda System for Reporting Thyroid Cytopathology (BSRTC) in study population. ROM = risk of malignancy; AUS/FLUS = Atypia of Undetermined Significance/Follicular Lesion of Undetermined Significance; sFN = suspicious for follicular neoplasm/follicular neoplasm; sM = suspicious for malignancy.

Bethesda Diagnosis	n	Benign	Malignant	ROM (%)	% 0f Total FNABs
Unsatisfactory	15	13	2	13.3	5.2
Benign	76	73	3	3.9	26.1
AUS/FLUS	65	48	17	26.2	22.3
sFN	54	40	14	25.9	18.6
sM	22	3	19	86.4	7.5
Malignant	59	1	58	98.3	20.3
Total	291	178	113	38.7	100.0

## Results

A total of 3307 thyroid FNABs were performed and interpreted at our institution between May 2010 and December 2014. Most of the FNABs were interpreted as being either benign (62.1%) or unsatisfactory (23.0%). There were 283 (8.6%) AUS/FLUS cases diagnosed, 95 (2.8%) sFN cases diagnosed, 54 sM cases diagnosed (1.6%), and 60 cancers (2.2%) diagnosed. The overall proportion of the study population with an indeterminate BSRTC diagnosis was 11.1%. Of the total number of individuals undergoing FNAB at our center, 291/3307 (8.8%) also underwent a thyroid operation at our center. Of these 291 cases, 15 (5.2%) FNABs were unsatisfactory, 76 (26.1%) were benign, 65 (22.3%) were AUS/FLUS, 54 (18.6 %) were sFN, 22 (7.5%) were sM and 59 (20.3%) were malignant (Table 1). Of the 65 AUS/FLUS cases, 11 underwent repeat FNAB. Nine of the repeat FNABs were again diagnosed as AUS/FLUS, and two were benign. Overall, of the 291 operated cases, 113 were diagnosed as cancer by histopathology, making the overall rate of malignancy in the study population 38.8%. The majority of the thyroid cancer cases were diagnosed by final histopathology as being PTC (101/113, 89.4%) and the remaining 12 (10.6%) cases were follicular carcinomas (FTC). Of the PTC cases 11 (10.9%) were subcategorized as the follicular variant of PTC. During the study period, there were two anaplastic carcinoma cases and that were diagnosed by FNAB. These cases were excluded from the study population in order to eliminate a potential source of bias, as they were all readily diagnosed by preoperative cytological assessment.

In our study population there were 227 females and 64 males, and the mean study patient age was 50 years (range 20–83 years). There were 79 of 225 females (35%) who had a cancer, as diagnosed by histopathology. The mean age for females was 50 years (range 20–83 years), and for males was also 50 years (range 23–80 years). The rate of malignancy for each BSRTC category in females was; 14.2%, 3.2 %, 26.4 %, 19.5 %, 81.3% and 100% for non-diagnostic, benign, AUS/FLUS, sFN, sM, and malignant diagnoses, respectively (Table 2). There were 34 of 64 males (53.1%) who had a cancer diagnosed by postoperative histopathology. The rate of malignancy for each BSRTC diagnostic category in males was 0%, 7.7%, 25%, 46.2%, 66.7% and 95% for non-diagnostic, benign, AUS/FLUS, sFN, sM and malignant diagnoses, respectively (Table 2). Even though thyroid cancer was significantly more common in males (p=0.021), gender did not significantly influence specific BRSTC diagnostic category cancer risk estimation (Table 2).

**Table 2:** Gender comparison of the rates of thyroid malignancy ROM = risk of malignancy; AUS/FLUS = Atypia of Undetermined Significance/Follicular Lesion of Undetermined Significance; sFN = suspicious for follicular neoplasm/follicular neoplasm; sM = suspicious for malignancy.

Bethesda Diagnosis	Females (n)	Benign	Malignant	ROM (%)	Males (n)	Benign	Malignant	ROM (%)	P-value
Unsatisfactory	14	12	2	14.2	1	1	0	0	0.685
Benign	63	61	2	3.2	13	12	1	7.7	0.488
AUS/FLUS	53	39	14	26.4	12	9	3	25	0.9198
sFN	41	33	8	19.5	13	7	6	46.2	0.0561
sM	16	3	13	81.3	6	2	4	66.7	0.47
Malignant	40	0	40	100	9	1	8	88.9	0.143
Total	227	148	79	34.8	64	32	32	50.0	0.0297

Of the 291 patients that made up our study population 178 were younger than 55 years (45.5%) and 113 were 55 years or older (26.5%) (Table 3). The risk of malignancy in the non-diagnostic category was 20% for patients younger than 55 years and 0% for older patients. In the benign category, the risk of malignancy for the younger category was 5.6% vs 2.5% in the older category. In the AUS/FLUS category, the risk of malignancy was 36.8% in the younger category vs 7.4% in the older category. In the suspicious for follicular neoplasm category, the risk of malignancy in the younger group was 14.3% vs 42.1% in the older group. In the suspicious for malignancy category, the risk of malignancy was 92.3% in the younger group and 77.8% in the older group. In the malignant category, the risk of malignancy in the younger group was 100% vs. 92.3% in the older group. The overall risk of malignancy for patients younger than 55 years was 45.5%, and for patients 55 years or older was 26.5%. The difference in risk of malignancy between these two age groupings was statistically significant (p=0.0013). The malignancy risk was higher in the group of patients younger than 55 years. For the AUS/FLUS BSRTC diagnostic group, the cancer risk was significantly higher for patients younger than 55 years (p=0.0082) (Table 3).

**Table 3:** Rates of thyroid malignancy comparing ages 55 years. ROM = risk of malignancy; AUS/FLUS = Atypia of Undetermined Significance/Follicular Lesion of Undetermined Significance; sFN = suspicious for follicular neoplasm/follicular neoplasm; sM = suspicious for malignancy.

Bethesda Diagnosis	< 55 Years	Benign	Malignant	ROM (%)	>55 Years	Benign	Malignant	ROM (%)	P-values
Unsatisfactory	10	8	2	20	5	5	0	0	0.5238
Benign	36	34	2	5.6	40	39	1	2.5	0.6006
AUS/FLUS	38	24	14	36.8	27	25	2	7.4	0.0082
sFN	35	30	5	14.3	19	11	8	42.1	0.0427
sM	13	1	12	92.3	9	2	7	77.8	0.5442
Malignant	46	0	46	100	13	1	12	92.3	0.2203
Total	178	97	81	45.5	113	83	30	26.5	0.0013

## Discussion

At our Canadian center, over 700 thyroid FNABs are carried out and evaluated annually. Fortunately, most of the diagnoses are benign, which is consistent with reports from other institutions [4, 9]. Since the implementation and routine utilization of the BSRTC at our center in 2010, a subset of the total FNABs (13.6%) has been diagnosed as being indeterminate (AUS/FLUS or sFN or sM). Of these cases, our overall AUS/FLUS utilization rate was 8.3%, which is marginally higher than the recommended 7% utilization for this BSRTC diagnostic category [4]. An especially challenging diagnosis, AUS/FLUS has a wide range of variability in utilization across institutions, and it has been suggested that the ROM associated with this diagnostic category is actually higher than the quoted 5-15%, being more in the 20-25% range [3-4, 7]. A meta-analysis reported by our group found that for 291 patients with thyroid lesions that underwent operation, the overall ROM for an AUS/FLUS diagnosis was 26.8% [10].

The clinical management of the AUS/FLUS diagnostic group has represented an ongoing challenge. Even though many cases with an AUS/FLUS BSRTC diagnosis undergo a repeat FNAB, there has also been much research that has focused on the application of adjunctive molecular testing for facilitating appropriate clinical management. Such strategies add significant complexity and cost to patient management. However, age is an easily accessible patient characteristic that has received little attention in the literature with regards to its influence on patient ROM within the BSRTC diagnostic categories. In the current study, all patients were stratified into two groups, those younger than 55 years, and those 55 years or older, a cut-off point we selected because it also represents an important thyroid cancer clinical prognosticator that has been recently incorporated into the eighth edition of the American Joint Committee on Cancer (AJCC) Differentiated Thyroid Cancer Staging System [8]. We observed a statistically significant difference in the ROM in patients with an AUS/FLUS diagnosis who were younger than 55 years, with the ROM being 36.8% in the younger group, compared to 7.4% in the older group. This suggests that thyroid nodules with an AUS/FLUS diagnosis, although being more common and generally having a worse prognosis when diagnosed as malignancy in older people, tend to have a higher risk of actually being diagnosed as cancer when detected in younger people. Other groups have also made similar observations [11-12]. Thus, when utilizing the BSRTC, patient age should be taken into account in order to facilitate a more accurate estimate of their actual ROM. Recently, when used in conjunction with other clinical parameters such as nodule size, and ultrasound characteristics, patient age has been found to be a predictive factor for malignancy in patients younger than 65 years [12].

With regards to gender, although we observed a significantly higher overall cancer risk in males, similar to observations made by other groups [7, 11], our study population is relatively small (291 cases) limiting our ability to study its interaction with age, though further investigation seems warranted.

The limitations of our study include a selection bias that exists because only the cases that went on to undergo an operation at our center were included in the study population. This leads to a higher than expected ROMs in the unsatisfactory, benign and AUS/FLUS groups, when compared to their expected BSRTC ROMs [4]. Surgically resected AUS/FLUS nodules have actually been reported to have a ROM that ranges between 6% and 48% [4] as highlighted in the current ATA guidelines. Similarly, a meta-analysis that was reported by our group compared 13 different centers following the implementation of the BSRTC and reported a ROM for AUS/FLUS nodules to be ranging between 19–38% [10].

Despite FNAB being the critical diagnostic test for thyroid nodules, decisions regarding the extent of thyroid surgery are also influenced by many other clinical factors. With regards to individual BSRTC diagnostic categories, the false negative rate in the benign category was low, and only marginally higher than the expected BSRTC ROM. The false negatives in this group were due to a follicular carcinoma case and a single follicular variant of PTC case. With respect to the AUS/FLUS diagnostic group, five of 17 (29.4%) cancer cases were diagnosed as follicular variant of PTC. In the malignant diagnostic group, 58/59 cases (98%) were malignant by final histopathological assessment. The single case within this group that had a benign pathological diagnosis was described as a ‘follicular adenoma with cytological atypia’ and this diagnosis was confirmed by an external pathology review. Despite the overlap in observations made across several studies, there has been the suggestion that the risk of malignancy in the AUS/FLUS category may be institution-specific due to variability in its utilization and application by cytopathologists [4]. As the majority of thyroid lesions do not undergo an operation, there is really no consistent way to confirm whether or not many of the FNAB diagnoses were actually accurate. This is also true for the recently described benign pathological diagnosis: non-invasive follicular tumor with PTC-like nuclear features (NIFTP). Though reclassifying some of our malignant cases into the NIFTP category would decrease the ROM across all BSRTC groups, NIFTP is a histopathological diagnosis, and would not assist with cytology-based preoperative decision making. However, given efforts to clearly define NIFTP diagnostic criteria, along with its utilization in clinical practice, it is probable that the overall risks of malignancy across all BSRTC categories will decrease. Furthermore, a study limitation arises because some of the cases, especially those with an unsatisfactory, benign or AUS/FLUS BSRTC diagnosis may only be followed clinically, and because they did not undergo an operation, they are not included in our study population. In real-world practice, patient characteristics such as a history of prior neck radiation exposure, or a familial thyroid cancer history, influence the decision to operate and the extent of surgery, and this information is usually not available to reporting cytopathologists. Certainly, study of these variables, in combination with the BSRTC diagnosis, could potentially further augment cancer ROM estimation, and influence treatment.

In the current study, we found the ROM to be significantly higher in the AUS/FLUS category for patients younger than 55 years of age. However, the ROM presented across many studies, including our own, depends heavily upon the prevalence of thyroid cancer in the specific patient population being studied [13-14]. Thus, it is of paramount importance for each institution that employs the BSRTC to guide clinical decision making to monitor its usage and to be aware of their ROM for each individual diagnostic category [13].

## Conclusions

As our observations have demonstrated, the ROM for the AUS/FLUS BSRTC category is closer to the ROM of the sFN category in patients younger than 55 years. From a clinical perspective, these observations suggest that it may be appropriate for patients younger than 55 years of age with an AUS/FLUS diagnosis to be offered a diagnostic thyroid lobectomy, as opposed to observation or to a repeat FNAB, as recommended by the BSRTC. Irrespective of potential developments in this field, we believe that further study of the relationship between patient age, gender, and ROM within the BSRTC indeterminate diagnostic groups is warranted, and ultimately may lead to improved cancer risk estimation, and thus, better tailoring of treatment for individuals diagnosed with thyroid nodules.
